# Supplementary material for: Microwave quantum heterodyne sensing using a continuous concatenated dynamical decoupling protocol
Source: Nat Commun. 2025 May 12;16:4380. doi: 10.1038/s41467-025-59148-9 (PMC12069688; doi:10.1038/s41467-025-59148-9)
Supplement: Supplementary file 1 — Supplementary Information [file 41467_2025_59148_MOESM1_ESM.pdf]

Supplementary Information: Microwave quantum heterodyne sensing using a continuous concatenated dynamical decoupling protocol

Charlie J. Patrickson[1,*], Valentin Haemmerli[1], Shi Guo[1], Andrew J. Ramsay[2], Isaac J. Luxmoore[1]

[1]Department of Engineering, University of Exeter, EX4 4QF, UK.
[2]Hitachi Cambridge Laboratory, Hitachi Europe Ltd., CB3 0HE,UK.
[*]corresponding author: Charlie J. Patrickson (cp728@exeter.ac.uk).

Supplementary Note 1: V_B^- Coherence Protection

A key benefit of the CCDD scheme is that it enhances spin coherence. This provides a larger contrast at longer MW pulsewidths, increasing $\max|\frac{\partial(\Delta C)}{\partial B}|$ and therefore improving amplitude sensitivity. In this section we quantify these enhancements.

We start by characterizing the natural lifetimes of the spin system. In Fig. 1(a) we record a conventional Rabi oscillation. Fitting to a damped sine yields a coherence time of $T_{Rabi} = 36$ ns for a Rabi frequency of $\Omega = 100$ MHz. This short coherence time is a consequence of electron-nuclear spin interactions [1]. In Fig. 1(b) we record the longitudinal relaxation time, finding $T_1 = 14.9$ μs. To understand the effect of the CCDD drive relative to these natural coherence times, in Supplementary Fig. 1(c) we present CCDD driven Rabi oscillations. The coherence time is a function of the drive phase, θ_m, drive amplitude, Ω (which we fix at 100 MHz in all experiments), second drive amplitude, ϵ_m, and signal amplitude, g_x. In a typical CCDD implementation we find $T_{CCDD}(\theta_m = 0,\ \epsilon_m = 10\,\text{MHz},\ g_x = 0) = 7.477$ μs, in excellent agreement with the $T_2 \approx \frac{1}{2}T_1$ limit proposed for systems limited by two-phonon induced spin relaxation [2]. Changing $\theta_m = \pi/2$, we find $T_{CCDD}(\theta_m = \pi/2,\ \epsilon_m = 10\,\text{MHz},\ g_x = 0)$ $= 716$ ns. Whilst the coherence time is reduced compared to $\theta_m = 0$, it is ≈ 20 times larger than the unprotected spin system. Moreover application of a resonant signal largely recovers coherence, with $g_x = 2$ MHz we find the coherence is extended to $T_{CCDD}(\theta_m = \pi/2,\ \epsilon_m = 10\,\text{MHz},\ g_x = 2\,\text{MHz}) = 3.94$ μs. Supplementary Fig. 1(d) provides a more detailed view of the data and fits between 900 - 1000 ns.

To better understand the limits of the the coherence with and without a signal, we perform sweeps of the modulation amplitude, ϵ_m and signal amplitude, g_x. The coherence times are extracted from fits to the linewidth of the FFT of the Rabi oscillation and plotted in Supplementary Figure 1(e) and (f). As the modulation amplitude ϵ_m increases, we observe an initial sharp increase in coherence time, with a maximum $T_{CCDD}(\theta_m = \pi/2,\ \epsilon_m = 12\text{ MHz},\ g_x = 0) \approx 1150$ ns (Supplementary Figure 1(e)). Further increasing ϵ_m degrades the coherence, consistent with previous observations[3]. The origin of the dip in coherence time for modulation amplitudes centered around 8 MHz is unknown, but could be due to hyperfine coupling

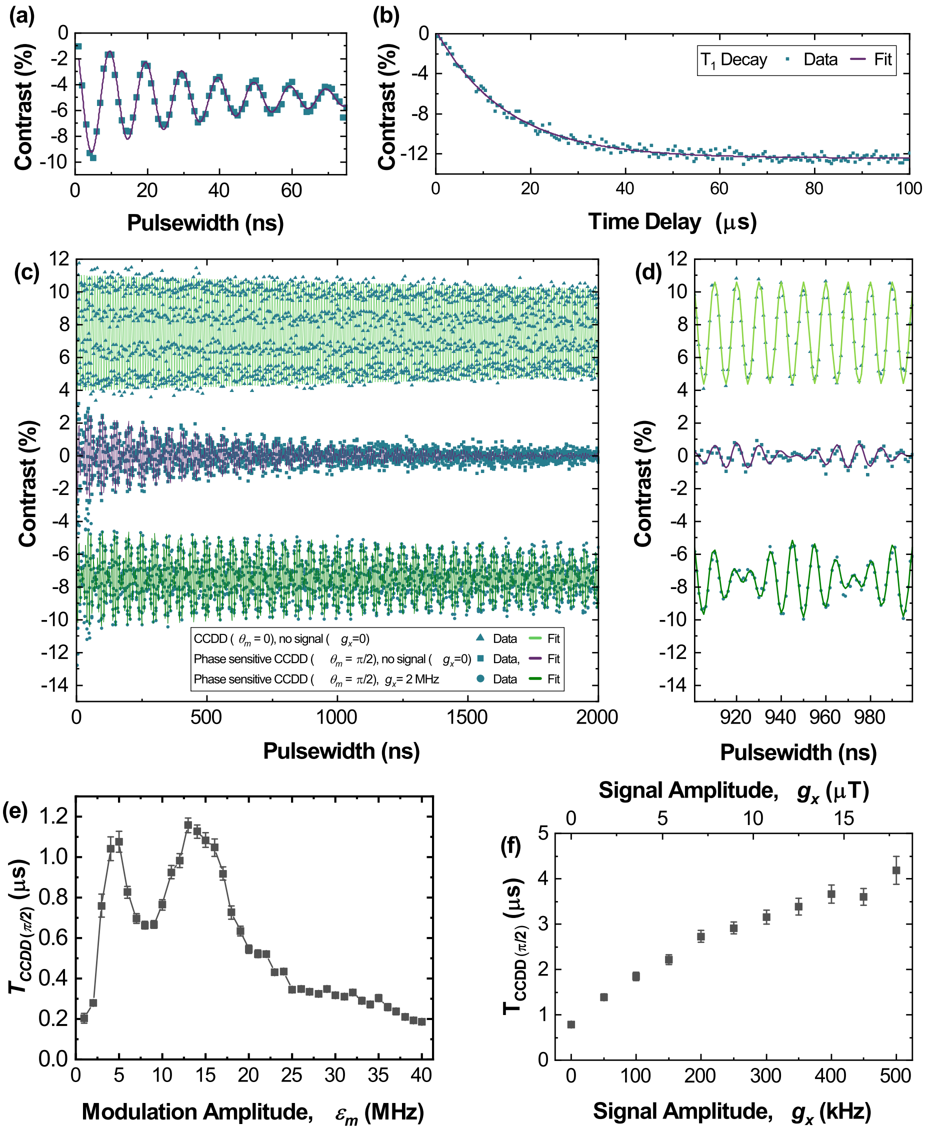

Supplementary Figure 1: V_B^- coherence times (a) Conventional Rabi oscillation of the $m_s = 0$ to $m_s = -1$ ground state transition. A fit to a damped sine $y = y_0 + y_1 \sin(\omega t + \phi)e^{-t/T_{Rabi}}$ gives a coherence time of $T_{Rabi} = 36$ ns.(b) T_1 decay, fitting to a damped exponential $y = y_0 + y_1 e^{-t/T_1}$ gives $T_1 = 14.9$ μs. (c) CCDD Rabi oscillations. In the absence of a signal field, choosing $\theta_m = 0$ ($\theta_m = \pi/2$) gives a fitted coherence time of $T_{CCDD}(\theta_m = 0,\ \epsilon_m = 10\,\text{MHz},\ g_x = 0) = 7.477$ μs, plotted in light green ($T_{CCDD}(\theta_m = \pi/2, \epsilon_m = 10\,\text{MHz}, g_x = 0) = 716$ ns, plotted in purple). A $g_x = 2$ MHz resonant signal extends the $\theta_m = \pi/2$ coherence time to $T_{CCDD}(\theta_m = \pi/2, \epsilon_m = 10\,\text{MHz}, g_x = 2\,\text{MHz}) = 3.94$ μs, plotted in dark green. $\theta_m = 0$ data is fit to $y = y_0 + y_1 \sin(\omega t + \phi)e^{-t/T_{CCDD}(\theta_m=0,\ \epsilon_m,\ g_x)}$, while $\theta_m = \pi/2$ data is fit to $y = y_0 + (y_1 \cos(\omega_1 t + \phi_1) + y_2 \cos(\omega_2 t + \phi_2))e^{-t/T_{CCDD}(\theta_m=\pi/2,\ \epsilon_m,\ g_x)}$.(d) Close up of (c) between 900 and 1000 ns. (e) $T_{CCDD}(\theta_m = \pi/2,\ \epsilon_m,\ g_x = 0)$ as a function of modulation amplitude, ϵ_m, in the absence of a signal (g_x=0). (f) $T_{CCDD}(\theta_m = \pi/2, \epsilon_m = 10\,\text{MHz}, g_x)$ as a function of signal amplitude, g_x, for a fixed modulation amplitude, $\epsilon_m = 10$ MHz. In (e) and (f) each data point corresponds to the linewidth of a lorentzian fit to the FFT of the CCDD Rabi oscillation. The error bars indicate the uncertainties obtained from the fitting procedure.

to neighboring nuclei[4, 5]. In the case of a resonant signal, plotted in Supplementary Figure 1(f), we observe a monotonic increase in the coherence time, reaching $T_{CCDD}(\theta_m = \pi/2,\ \epsilon_m = 10\,\text{MHz},\ g_x = 400\,\text{kHz}) \approx 4\ \mu$s.

Supplementary Note 2: Device Response to Signal Frequency

In this section we start by discussing the sensor response to off-resonant signals. We perform CCDD Rabi oscillations analogous to Fig. 2 of the main text, but as a function of signal frequency. The interaction is illustrated by the FFTs plotted in Supplementary Fig. 2(a) and (b) for signal phases of $\phi_s = 0$ and $\frac{\pi}{2}$, respectively. The Fourier response centres on the same nested Mollow triplet structure as in the main text, with a central frequency of $\Omega = 100$ MHz, CCDD sidebands at $\Omega \pm \epsilon_m = 100 \pm 10$ MHz and signal induced sidebands. The latter is complex in structure, and depends on the detuning between the sensor resonance and the signal frequency, $\delta = \omega_s - \Omega_0 - \epsilon_m$. The sensor undergoes a sharp transition when exposed to a resonant signal, shown here at a signal frequency of 2.31 GHz, with distinct responses for each signal phase. In particular, the main frequency components change from $\Omega = 100\,\text{MHz}$ to $\Omega \pm \epsilon_m = 100 \pm 10\,\text{MHz}$ for signal phases of $\phi_s = 0$, and from $\Omega \pm \epsilon_m = 100 \pm 10\,\text{MHz}$ to $\Omega \pm g_x = 100 \pm 1\,\text{MHz}$ for signal phases of $\phi_s = \frac{\pi}{2}$. This illustrates our protocol's dependence on signal frequency, which we detect by effectively filtering between these different Fourier regimes. Far from resonance, the response reduces to frequencies at $\Omega \pm \epsilon_m$, as expected for a CCDD Mollow triplet with the drive phase $\theta_m = \frac{\pi}{2}$ [4].

The CCDD microwave drive also produces multiple other sensor resonances. Each can be tuned using the drive parameters and display a similar response [6]. This is an important feature of CCDD sensing schemes, as tuning the electron spin transition normally involves changing the DC magnetic field by moving an external magnet, which is slow and imprecise. Whereas in our device, the sensor resonances can be

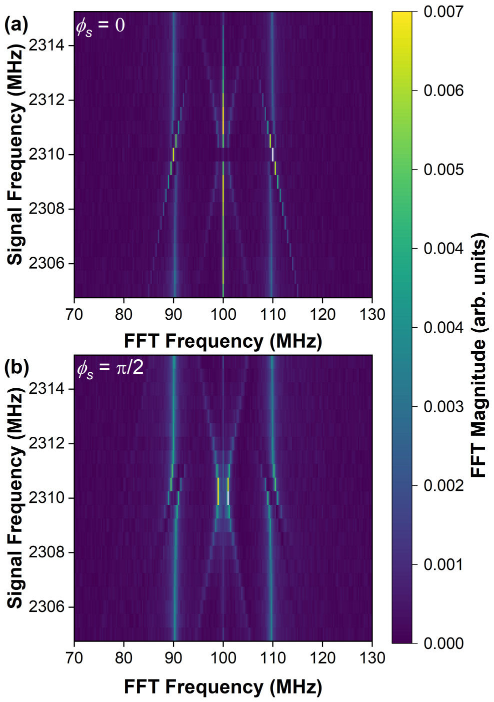

Supplementary Figure 2: Off resonant sensor response. Fourier transforms of CCDD driven Rabi oscillations as a function of applied signal frequency for signal phases of $\phi_s = 0$ in **(a)** and $\phi_s = \frac{\pi}{2}$ in **(b)**. The sensor resonance is set to $\omega_s = \omega_0 - \epsilon_m = 2.31$ GHz. When this condition is met a sharp change in the Fourier response is seen for both signal phases, indicating the device sensitivity to signal frequency. Further information can be found in [6]

tuned within ±150 MHz of the electron spin transition electronically, as illustrated in Supplementary Fig. 3. This is useful when probing signals of unknown frequency.

Using our protocol, in Supplementary Fig. 4 we show that these additional resonances can also resolve signal phase. We use the same CCDD parameters as those in the main text, which targeted a resonance at $\omega_s = \omega_0 - \epsilon_m = 2.31$ GHz. Instead, here we apply a signal frequency of $\omega_s = \omega_0 - \Omega + \epsilon_m = 2.23$ GHz and analyse the response of a CCDD Rabi measurement. To illustrate the dependence on signal phase, Supplementary Fig. 4(a) shows the response for $\phi_s = 0$ and $\frac{\pi}{2}$. The Fourier response is presented in Supplementary Figs. 4(b), where it is clear that each signal phase produces a different sensor output, analogous to the results presented in the main text. Note that the Fourier response for each signal phase is reversed in comparison to the

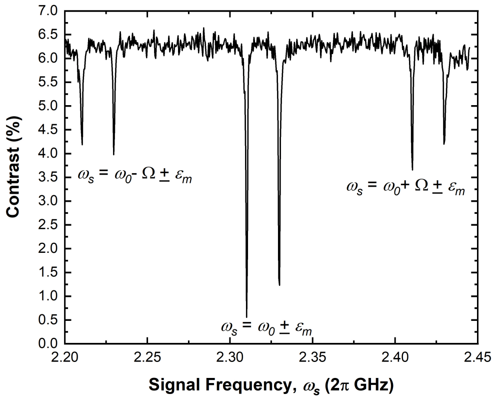

Supplementary Figure 3: Sensor resonances Sensor response as a function of signal frequency ω_s, displaying six sensor resonances centered on the electron spin transition, $\omega_0 = 2.32$ GHz. The contrast was sampled after exposing the V_B^- ensemble to a CCDD pulsewidth of $T_{MW} = 950$ ns. The applied signal had an amplitude of $g_x = 0.8$ MHz and phase $\phi_s = 0$. Drive phase was omitted, $\theta_m = 0$.

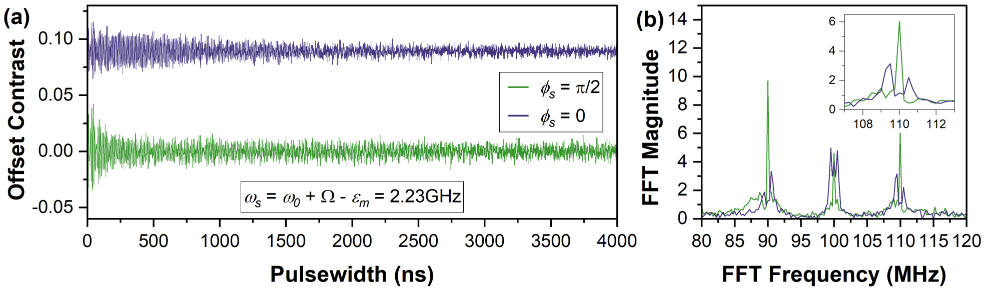

Supplementary Figure 4: Alternative sensor resonance. (a) CCDD driven Rabi oscillation with the same parameters as in the main text, but for a signal frequency of $\omega_s = 2.23$ GHz instead of $\omega_s = 2.31$ GHz. The two resonances display the same behaviour as in the main text, demonstrating that the additional tuneable resonances produced through the CCDD scheme are also sensitive to signal phases of $\phi_s = 0$ (blue) and $\phi_s = \pi/2$ (green). **(b)** Fourier transform of (a). The inset shows a closeup of the right hand Mollow triplet, which presents an opposite phase response to the resonance used in the main text.

resonance in the main text. This has no impact on the sensors ability to distinguish signal phase as the protocol only requires two distinct responses to contrast against. We also note that the magnitude of the Fourier response is reduced for signal frequencies of $\omega_s = \omega_0 - \Omega + \epsilon_m = 2.23$ GHz. This applies for all resonances other than $\omega_s = \omega_0 \pm \epsilon_m$, as they are subject to increased attenuation in the dressed state frame of reference [7].

Supplementary Note 3: Spin Response to Signal Vector

CCDD sensing schemes operate by driving the spin vector along multiple axes at multiple frequencies, producing additional resonances in the system whilst also decoupling the spin from sources of magnetic noise. These dynamics produce a spin response that depends simultaneously on the signal frequency and its direction of propagation. Our sensor displays six resonances centred on the electron spin resonance. The frequencies depend on the CCDD drive amplitudes Ω and ϵ_m and are only sensitive to signals propagating in the XY plane[6]. Two additional resonances appear in the MHz range, which are sensitive to signals propagating along the z-axis, and depend only on Ω and ϵ_m. In the main text we focused on a single resonance for a signal propagating along the x-axis. Here we consider three signals, one propagating along each axis. Selecting a single resonance for each we show that the protocol retains phase sensitivity regardless of the signal direction.

We consider the signal field,

$$H_s = (g_x\sigma_x + g_y\sigma_y + g_z\sigma_z)\cos(\omega_s t + \phi_s), \tag{1}$$

where g_x, g_y and g_z are the signal amplitudes along each axis, σ_i are the Pauli operators, ω_s is the signal frequency and ϕ_s is signal phase. We move through two rotating reference frames, first with respect to $\frac{1}{2}\omega_0\sigma_z$, and then $\frac{1}{2}\omega_m\sigma'_x$ (primes denote the reference frame)[6], and select a single sensor resonance for each axis of propagation;

$$H''_{s,x} = \frac{1}{2}g_x\sigma''_x\cos((\omega_s - \omega_0)t + \phi_s), \tag{2}$$

$$H''_{s,y} = -\frac{1}{2}g_y\sigma''_y\sin((\omega_s - \omega_0)t + \phi_s), \tag{3}$$

$$H''_{s,z} = \frac{1}{2}g_z\sigma''_z(\cos((\omega_s - \omega_m)t + \phi_s) - \sin((\omega_s - \omega_m)t + \phi_s)), \tag{4}$$

where ω_0 describes the electron spin resonance and we choose $\omega_m = \Omega$ to meet the conditions of the CCDD scheme. Choosing $\epsilon_m = \omega_s - \omega_0$ for Supplementary Eqs. 2

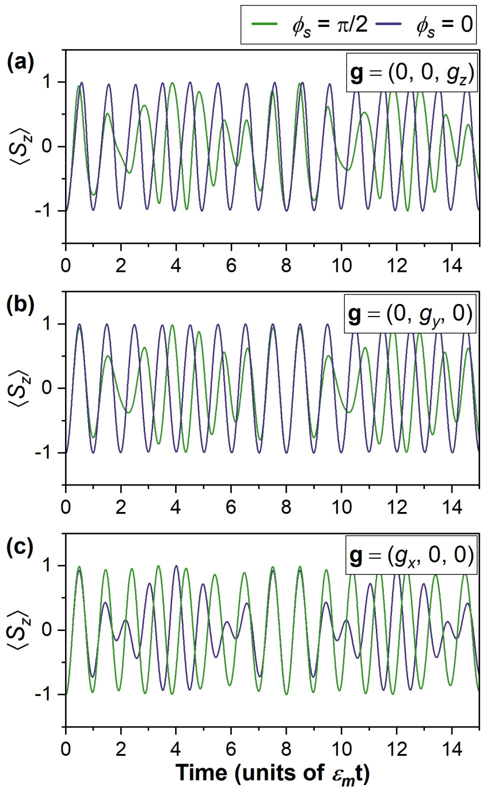

Supplementary Figure 5: Rotating frame spin dynamics modelled under different signal vectors. Z projection of the rotating frame spin vector as a function of time, modelled for a signal vector of $\mathbf{g} = (0, 0, g_z)$ and signal frequency of $\omega_s = \Omega + \epsilon_m$ in **(a)**, $\mathbf{g} = (0, g_y, 0)$ and signal frequency of $\omega_s = \omega_0 + \epsilon_m$ in **(b)** and $\mathbf{g} = (g_x, 0, 0)$ and signal frequency of $\omega_s = \omega_0 + \epsilon_m$ in **(c)**. Modelled for signal amplitudes of $g_{x,y,z} = \frac{1}{4}\epsilon_m$ and signal phases of $\phi_s = 0$ (blue) and $\phi_s = \frac{\pi}{2}$ (green).

and 3, and $\epsilon_m = \omega_s - \omega_m$ for Supplementary Eq. 4 satisfies the resonance conditions of the sensor. Our aim is to demonstrate that for each signal in Eqs. 2, 3 and 4, the sensor response will depend on the signal phase ϕ_s. To illustrate this we model the spin evolution for each signal when driven by the CCDD field. Damping effects are not considered. As the sensor readout projects the spin onto the z-axis, in Supplementary Fig. 5 we plot the z-component of the spin vector as a function of time, for signal phases of $\phi_s = 0, \frac{\pi}{2}$ and signals along the z, y and x axes in (a), (b) and (c) respectively. For each signal, the spin has a phase-dependent response, illustrating the protocols ability to resolve signal phase for any signal vector.

Supplementary Note 4: Device Response to Signal Amplitude

In the Fourier domain, the frequencies of the nested Mollow triplet structure are determined by the field amplitudes of the CCDD microwave drives, Ω and ϵ_m, and the signal, g_x. This means that, for a fixed set of CCDD amplitudes, small changes in the signal amplitude g_x will change the Rabi frequencies of the sensor. Monitoring the contrast at a fixed point in the Rabi oscillation provides a way of detecting any changes to the Rabi frequency - and thereby the signal amplitude. If the fixed point is selected appropriately, changes in signal phase and amplitude produce opposite responses, such that changes in the two can be distinguished (see Fig. 3(a) of the main text). Supplementary Fig. 6 plots the Mollow triplet structure as a function of signal amplitude, for a signal phase of $\phi_s = \frac{\pi}{2}$. The Fourier components centre on the CCDD drive fields $\Omega = 100$ MHz and $\Omega \pm \epsilon_m = 100 \pm 10$ MHz. The significant Fourier components are offset from these central values by the signal amplitude, to $\Omega \pm g_x = 100 \pm g_x$ MHz and $\Omega \pm \epsilon_m \pm g_x = 100 \pm 10 \pm g_x$ MHz. This manifests as diverging Fourier components in Supplementary Fig. 6, illustrating the devices sensitivity to signal amplitude.

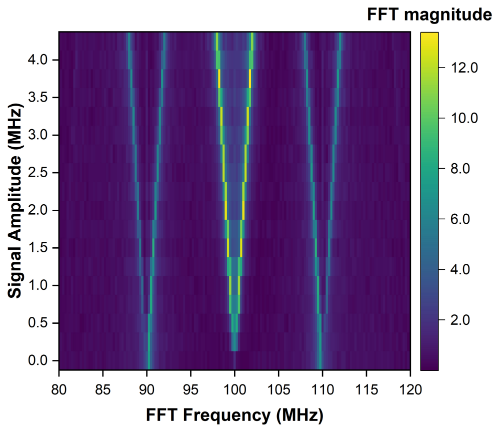

Supplementary Figure 6: Fourier response to signal amplitude Mollow triplet structure as a function of signal amplitude. Each Fourier transform was produced from a Rabi oscillation recorded over 4000 ns, for a fixed signal amplitude. The signal phase was $\phi_s = \frac{\pi}{2}$ at a frequency $\omega_s = 2.31$ GHz. The divergent Fourier components illustrate the devices sensitivity to signal amplitude.

Supplementary Note 5: Optimised Heterodyne Measurement Time

The quantum heterodyne detection protocol presented in the main text tracked the evolution of signal phase across successive readouts, with each lasting 2.5 μs. Note that it is beneficial to minimise the length of this sequence. Firstly, it enables a higher sampling resolution, which improves the SNR and increases the Fourier resolution. It also increases the Nyquist frequency of the heterodyne protocol, increasing the frequency range that can be detected for a fixed set of CCDD parameters. The 2.5 μs sequences consisted of 1.35 μs of optical initialisation/ readout, 950 ns of CCDD drive with 100 ns of idle time either side. This relatively long CCDD drive time maximised the amplitude sensitivity, as it provided more time for weak signals to drive a detectable change in spin state. However, this can be dramatically shortened if amplitude detection is not required. To demonstrate phase sensitivity it is sufficient to differentiate between two Fourier responses which are largely defined by the drive amplitude ϵ_m for $\epsilon_m >> g_x$. This is illustrated by the Rabi sequence and associated Fourier transform in Supplementary Figs. 7(a) and (b), where the inset shows a departure in the sensors phase response on timescales of $\approx 1/(\epsilon_m - g_x) \approx 125$ ns. Results presented here used $\epsilon_m = 10$ MHz, however the CCDD drive functions with values of ϵ_m up to 25 MHz (see Supplementary Note 1 and Supplemetary Fig. 1), which could further reduce this time to $\approx 1/(\epsilon_m - g_x) \approx 40$ ns. At this limit each measurement sequence will be constrained by the optical initialisation and readout rate. Further optimisation could bring this down to 200 ns[8], to realise a total measurement sequence lasting 500 ns.

Supplementary Note 6: Signal Amplitude Dependence of Heterodyne signal to Noise Ratio

The coherence time $T_{CCDD}(\theta_m = \pi/2,\ \epsilon_m,\ g_x)$, and therefore the phase sensitivity, of the CCDD protocol is dependent on the signal amplitude, g_x. As this protocol

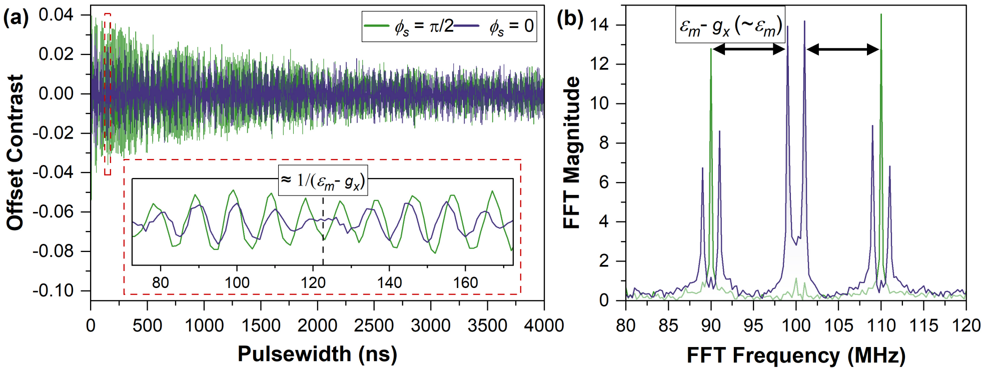

Supplementary Figure 7: Optimisation of heterodyne measurement time (a) CCDD Rabi oscillation exposed to a resonant signal with phases of $\phi_s = 0$ (blue) and $\phi_s = \frac{\pi}{2}$ (green). The sensor can differentiate between signal phases on timescales of $\approx 1/(\epsilon_m - g_x)$, shown in the inset. **(b)** Fourier transform of (a). Each signal phase produces a different Fourier response. For small signal amplitudes where $g_x << \epsilon_m$, the major Fourier components contributing to each response are largely determined by the drive parameter ϵ_m.

underpins the heterodyne measurements we expect to see a signal-to-noise ratio that depends on the signal amplitude. This is verified by the measurement shown in Supplementary Figure 8. As the signal amplitude increases to greater than ~ 10 μT the SNR increases as the signal provides additional coherence protection, and reaches a maximum when the signal amplitude at ~ 35 μT ≈ 1 MHz $\approx 1/T_{MW}$[6].

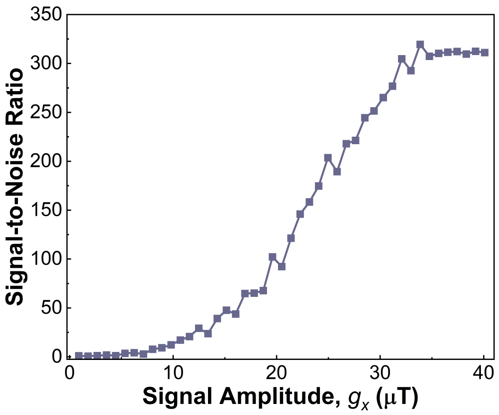

Supplementary Figure 8: Heterodyne signal-to-noise ratio as a function of the signal amplitude. The measurement parameters are the same as used in Fig. 4 of the main text and an integration time of 10 s.

Supplementary Note 7: Heterodyne Detection of Amplitude Modulated Signals

Classical heterodyne detection has historically been used to detect amplitude or frequency modulated signals used in communications protocols. In this section we demonstrate that our quantum heterodyne scheme can also detect complex signals composed of multiple signal frequencies for a single set of CCDD parameters. Using the heterodyne detection parameters presented for Fig. 4, we record the sensor response under exposure to a carrier wave detuned by 20 kHz from the spin resonance and amplitude modulated at 2 kHz. The FFT of the autocorrelated sensor output is plotted in SI Fig. 9, which records the carrier frequency at 40 kHz corresponding to twice the detuned value, sidebands at 40 ± 2 kHz and the amplitude modulated frequency of 2 kHz. A similar response is expected for frequency modulated signals or multiple independent signals provided the transmission frequencies fall within the 100 kHz range of the spin resonance.

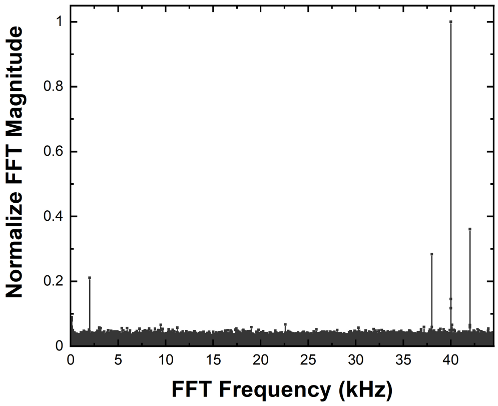

Supplementary Figure 9: Heterodyne Detection of an Amplitude Modulated Signal FFT of an autocorrelated heterodyne measurement for a 2 kHz amplitude modulated signal detuned by 20 kHz from the sensor resonance at $\omega_s = 2.32$ GHz. Four peaks are visible corresponding to the amplitude modulation at 2 kHz, double the carrier wave detuning at 40 kHz and two sidebands at 40 ± 2 kHz. Remaining measurement parameters are as described for Fig. 4. of the main text

Supplementary References

[1] Haykal, A. et al.: Decoherence of V_B^- spin defects in monoisotopic hexagonal boron nitride. Nature Communications **13**, 4347 (2022) https://doi.org/10.1038/s41467-022-31743-0

[2] Bar-Gill, N., Pham, L. M., Jarmola, A., Budker, D., Walsworth, R. L.: Solid-state electronic spin coherence time approaching one second. Nature communications **4**, 1743 (2013) https://doi.org/10.1038/NCOMMS2771

[3] Wang, G., Liu, Y. X., Cappellaro, P., Coherence protection and decay mechanism in qubit ensembles under concatenated continuous driving. New Journal of Physics **22**, 123045 (2020) https://doi.org/10.1088/1367-2630/abd2e5

[4] Ramsay, A. J. et al.: Coherence protection of spin qubits in hexagonal boron nitride. Nature Communications **14**, 461 (2023) https://doi.org/10.1038/s41467-023-36196-7

[5] Ivády, V. et al.: Ab initio theory of the negatively charged boron vacancy qubit in hexagonal boron nitride. npj Computational Materials **6**, 41 (2020) https://doi.org/10.1038/s41524-020-0305-x

[6] Patrickson, C. J., Baber, S., Gaál, B. B., Ramsay, A. J., Luxmoore, I. J.: High frequency magnetometry with an ensemble of spin qubits in hexagonal boron nitride. npj Quantum Information **10**, 5 (2024) https://doi.org/10.1038/s41534-023-00796-4

[7] Salhov, A., Cao, Q., Cai, J., Retzker, A., Jelezko, F., Genov, G.: Protecting quantum information via destructive interference of correlated noise. Physical Review Letters **132**, 223601 (2024) https://doi.org/10.1103/PhysRevLett.132.223601

[8] Clua-Provost, T. et al.: Spin-dependent photodynamics of boron-vacancy centers in hexagonal boron nitride. Physical Review B **110**, 014104 (2024) https://doi.org/10.1103/PhysRevB.110.014104

[9] Patrickson, C.J., Haemmerli V., Guo S., Ramsay, A. J. and Luxmoore I. J.: "Microwave quantum heterodyne sensing using a continuous concatenated dynamical decoupling protocol (dataset)", Open Research Exeter, https://doi.org/10.24378/exe.5687, 2025
